# All-Cause Mortality during First Wave of Pandemic (H1N1) 2009, New South Wales, Australia, 2009

**DOI:** 10.3201/eid1609.091723

**Published:** 2010-09

**Authors:** David J. Muscatello, Michelle A. Cretikos, C. Raina MacIntyre

**Affiliations:** Author affiliations: New South Wales Department of Health, North Sydney, New South Wales, Australia (D.J. Muscatello, M.A. Cretikos);; University of New South Wales, Kensington, New South Wales, Australia (D.J. Muscatello, C.R. MacIntyre);; University of Sydney, Sydney, New South Wales, Australia (M.A. Cretikos)

**Keywords:** Mortality, influenza, pandemic (H1N1) 2009, human, disease outbreaks, viruses, Australia, research

## Abstract

In temperate countries, death rates increase in winter, but influenza epidemics often cause greater increases. The death rate time series that occurs without epidemic influenza can be called a seasonal baseline. Differentiating observed death rates from the seasonally oscillating baseline provides estimated influenza-associated death rates. During 2003–2009 in New South Wales, Australia, we used a Serfling approach with robust regression to estimate age-specific weekly baseline all-cause death rates. Total differences between weekly observed and baseline rates during May–September provided annual estimates of influenza-associated death rates. In 2009, which included our first wave of pandemic (H1N1) 2009, the all-age death rate was 6.0 (95% confidence interval 3.1–8.9) per 100,000 persons lower than baseline. In persons >80 years of age, it was 131.6 (95% confidence interval 126.2–137.1) per 100,000 lower. This estimate is consistent with a pandemic virus causing mild illness in most persons infected and sparing older persons.

## Abstract 2

TOC Summary: Death rates from all causes were lower than in some recent influenza seasons, particularly among older persons.

Influenza epidemics can be associated with large increases in all-cause and cause-specific death rates (*1**–**4*). Increases have been observed in the rate of deaths attributed to influenza, pneumonia, circulatory disease, and diabetes (*4**,**5*). Many deaths in which influenza infection is a factor do not have laboratory confirmation of infection. Public health surveillance thus incorporates indirect measures of disease caused by influenza. Analyses based on the Serfling approach (*1*) frequently have been used to assess the effects of influenza epidemics on mortality time series (*2**,**3**,**6**–**10*). Because deaths increase in winter, even when influenza epidemics are not occurring, these models are required so that observed death rates during influenza epidemics can be differentiated from a seasonally changing baseline. The models typically do not incorporate virologic data but nevertheless produce estimates similar to models that do incorporate markers of specific virologic activity (*4**,**11*).

Although the first winter wave of pandemic (H1N1) 2009 in New South Wales, Australia, substantially increased demand for acute health services, impact on death from pneumonia and influenza (P&I) was limited, even in comparison with recent seasonal influenza epidemics (*12*). New South Wales is Australia’s most populous state (population 7.0 million) and includes Australia’s largest city and primary entry port, Sydney (4.4 million persons).

We applied a Serfling-based approach to time series of age-specific population rates of all-cause death rates for 2003–2009. Our analysis more completely assesses the impact of death temporally associated with the first wave of pandemic (H1N1) 2009 in New South Wales.

## Methods

For scheduled disease surveillance, death registrations from the New South Wales Registry of Births, Deaths and Marriages are provided daily to the New South Wales Department of Health and securely stored in the Health Outcomes and Information Statistical Toolkit database (*13*). The database also includes the Australian Bureau of Statistics (ABS) Cause of Death Data Collection (*14*), which is less current than the registry data source.

We assembled a time series of weekly rates of deaths per 100,000 population for 5 age groups (0–19, 20–49, 50–64, 65–79, and >80 years) for weeks ending on Fridays from January 1, 2003, through September 30, 2009. ABS death data were used for deaths occurring before January 1, 2006, and New South Wales Registry data were used for subsequent years. We calculated weekly death rates using estimated resident populations linearly interpolated from mid-year estimates (*15*) and projections (*16*).

To forecast the seasonally varying baseline time series of mortality that would be expected when influenza was not at epidemic levels, Serfling (*1*) used an ordinary linear regression model with terms that describe a consistent and cyclic winter rise and summer decline in mortality (harmonic terms). Before fitting the model, he removed observed values during influenza epidemics to prevent the baseline being raised by the epidemics. We adopted a similar approach, but we used robust regression, which does not require removal of observations during epidemic periods. This approach is used for seasonal P&I mortality surveillance by the US Centers for Disease Control and Prevention and the New South Wales Department of Health (*13**,**17*).

Briefly, robust regression is a model-fitting procedure that limits the influence of statistical outliers (extreme observations) on the resulting model. This model is important for influenza surveillance because influenza epidemics cause outliers in the time series, and the influence of influenza epidemics needs to be limited when background seasonal pattern is estimated. During cooler months, the difference between observed and baseline mortality can then be attributed to the impact of influenza on mortality.

For this analysis, we included a sequential week number in the model to capture long-term linear trend, the square of week number to capture any long-term curved trend, and harmonic terms with annual periodicity for the seasonal pattern. Categorical age group was included in the model. To enable the trend and seasonal terms to vary by age group, we also included terms for interactions between age group and each of the time variables (Technical Appendix).

To estimate annual differences between observed and expected death rates for each age group, we first calculated the difference between the observed all-cause death rate and the modeled baseline rate in each week during May–September. This 5-month period was sufficiently long to include seasonal influenza activity in most years and the time during which pandemic (H1N1) 2009 circulated during 2009. The weekly rate difference was applied to the interpolated population estimate for the month to obtain an estimated count difference for the week. These differences, whether positive or negative, were summed over all weeks during May–September to obtain total count differences for the year’s influenza season. We then divided the resulting total count difference by the average population during May–September of that year to obtain the total rate difference. Negative differences indicate that the observed death rates were lower than expected compared with the seasonal baseline.

To obtain crude all-age difference in death rates, we first calculated totals for age-specific observed deaths, baseline estimated deaths, and population estimates for each week in the study period. Confidence intervals (CIs) for the total of the predicted baseline rates were obtained (Technical Appendix). We then calculated the all-age rate and count differences and their CIs in the same way as the age-specific differences.

To permit an age-adjusted comparison between years, we also calculated standardized rates from the age-specific rates using the 2009 mid-year population as the standard. The upper 95% confidence limit of each year’s standardized rate was obtained by standardizing the upper 95% confidence limit of each of the age-specific rates. The equivalent procedure was followed to obtain the lower 95% confidence limit in each year.

For visual comparison, weekly P&I death rates per 250,000 population by age were calculated from the same death databases used for all-cause death rates. For the pre-2006 ABS vital statistics dataset, we included any death with an underlying or contributing cause of death in the International Classification of Diseases, 10th Revision, codes J09–J18. For the 2006–2009 registry dataset, we included any death registration with pneumonia or influenza mentioned anywhere in the causes of death using the same algorithm as our weekly P&I mortality surveillance (*13*). The rate per 250,000 rather than 100,000 population was presented to enable visual inspection of detail in the time series.

To compare death rates with influenza activity, the weekly proportion of respiratory specimens that tested positive for influenza was obtained from the New South Wales Department of Health’s routine influenza surveillance information reported by up to 8 major public pathology laboratories in New South Wales during the years studied. Reporting occurs from early May through the end of September each year. Testing was by direct immunofluorescence or PCR (*18*).

To assess consistency between observed mortality patterns and circulating influenza strains, subtyping information for New South Wales specimens was obtained from the Australian World Health Organization Collaborating Centre for Reference and Research on Influenza (Melbourne, Victoria, Australia) (A. Hurt, pers. comm.). In some years, 2 strains predominated, so we defined predominant strains as those identified in at least 33% of specimens in the year.

The death registration data files used for the study had identifying details, including names, addresses, and dates of birth, removed. Therefore, ethics approval was not required.

To assess the plausibility of our results, we repeated the analysis using 2 alternative methods. First, the analysis was rerun fitting a separate robust regression model to each age group without any age-specific parameters. Second, we applied a more traditional Serfling approach to our original age-specific analysis, which involved removing weekly rates during May–September and fitting an ordinary linear regression model without robust estimation. The methods are described in more detail in the Technical Appendix.

## Results

In New South Wales during January 1, 2003–September 30, 2009, median weekly all-cause death rates for persons 0–19 years of age were <1/100,000 in all years. For persons 20–49 years of age, they were ≈2/100,000; for persons 50–64 years of age, ≈10/100,000; for persons 65–79 years of age, ≈40/100,000; and for persons >80 years of age, ≈180/100,000 (Table 1).

**Table 1:** Weekly all-cause death rates per 100,000 population used in the regression model, New South Wales, Australia, January 2003–September 2009

Year	Median (interquartile range), by age group, y				
	0–19	20–49	50–64	65–79	>80
2003	0.8 (0.4)	1.9 (0.4)	9.3 (0.9)	43.3 (6.9)	187.0 (46.5)
2004	0.8 (0.3)	1.8 (0.3)	9.1 (1.6)	42.0 (4.9)	183.3 (50.6)
2005	0.8 (0.3)	1.8 (0.4)	8.7 (1.3)	39.6 (4.3)	176.5 (40.3)
2006	0.8 (0.3)	1.8 (0.4)	8.5 (1.4)	38.2 (4.7)	180.4 (29.1)
2007	0.7 (0.3)	1.8 (0.3)	8.9 (1.1)	37.9 (6.3)	179.0 (36.7)
2008	0.7 (0.4)	1.7 (0.3)	8.4 (1.3)	36.5 (4.5)	181.4 (40.1)
2009	0.7 (0.3)	1.8 (0.3)	8.6 (1.2)	35.2 (3.3)	175.1 (31.2)

**Figure A1:**
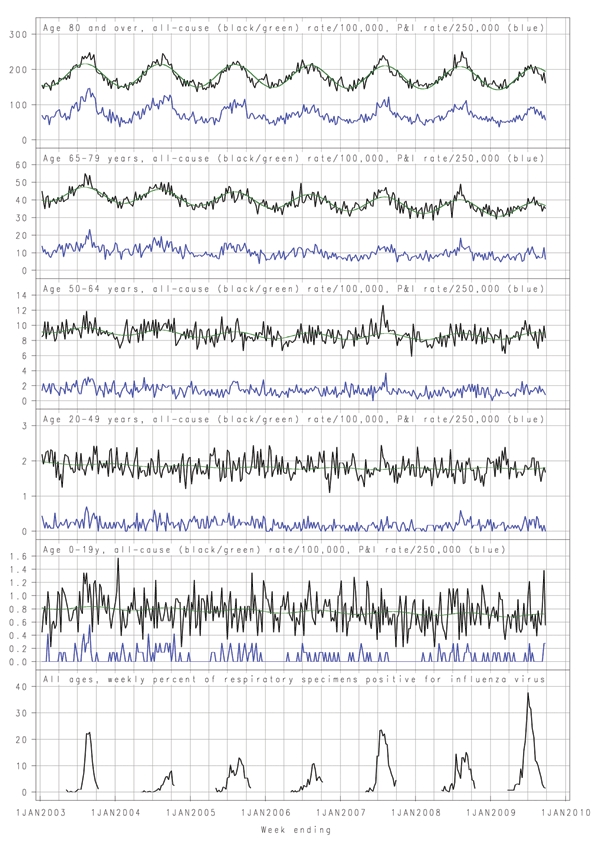
Weekly age-specific all-cause death rates per 100,000 population (black), fitted seasonal baseline (green), weekly pneumonia and influenza deaths (P&I) per 250,000 population (blue), and all-age weekly proportion of respiratory samples positive for influenza, New South Wales, Australia, January 2003–September 2009. The weekly proportion of respiratory samples positive for influenza was drawn from data provided by up to 8 major New South Wales public pathology laboratories for persons of all ages. Testing was by direct immunofluorescence or PCR. These data were reported only during May–September each year.

The seasonal fluctuations in mortality were more evident in the older age groups, and death rates in persons 65–79 years of age also declined steadily over the study period (Figure A1). Among persons >80 years of age, periods of increased death rates in the cooler months relative to baseline occurred in 2003, 2004, 2007, and 2008. These correspond to increases in P&I deaths and influenza circulation. Among persons 65–79 years of age, sustained peaks above baseline that correspond to influenza activity and peaks in P&I deaths are evident in 2003, 2007, and 2008. Among persons aged 50–64 years, a clear and consistent peak is evident in 2007, and possibly in 2009. Among persons 20–49 years of age, a peak is evident only in 2007, although the time series in that age group varied substantially. Among persons 0–19 years of age, no peaks clearly coincide with influenza activity. An increase coinciding with influenza circulation can be discerned in 2003, and a short-lived peak is evident in 2009, but some similar and larger peaks occurred at various times during the 7 years, not always when influenza was circulating (Figure A1).

Estimates of age-specific differences between observed and predicted baseline death rates for each year are shown in Table 2, and the all-age and age-standardized differences in Table 3. Predominant strains are also shown in Table 3, but no consistent association between antigenic characteristics and positive or negative rate differences is evident. In years with significant positive increases above baseline (those in which the 95% CI excludes zero), the magnitude of the difference increased dramatically with age (Table 2). Significant all-age increases in death rates above baseline occurred in 2003 with 10.0 (95% CI 7.3–12.6) deaths per 100,000 population more than expected; 2004 with 7.2 (95% CI 5.3–9.1) per 100,000 more; 2007 with 7.3 (95% CI 5.3–9.2) per 100,000 more; and 2008 with 8.6 (95% CI 6.5–10.6) per 100,000 more. Age-standardizing increased the all-age rate difference in 2003 and 2004 by ≈10% (Table 3).

**Table 2:** Difference between observed and baseline all-cause death rates and counts, New South Wales, Australia, January 2003–September 2009*

Age group, y, and year	Rate (95% CI)	No. (95% CI)
0–19		
2003	1.7 (–3.1 to 6.6)	31 (–56 to 117)
2004	–1.1 (–4.7 to 2.4)	–20 (–83 to 43)
2005	–1.0 (–4.7 to 2.8)	–17 (–84 to 50)
2006	–0.3 (–4.1 to 3.6)	–5 (–74 to 64)
2007	–0.8 (–4.5 to 2.8)	–15 (–81 to 51)
2008	0.8 (–3.0 to 4.6)	15 (–54 to 83)
2009	–0.1 (–5.5 to 5.4)	–2 (–100 to 97)
20–49		
2003	0.8 (–4.1 to 5.7)	23 (–118 to 164)
2004	–1.6 (–5.1 to 2.0)	–45 (–148 to 58)
2005	0.3 (–3.5 to 4.1)	9 (–101 to 119)
2006	1.7 (–2.2 to 5.5)	49 (–64 to 161)
2007	1.0 (–2.7 to 4.6)	29 (–79 to 136)
2008	0.2 (–3.6 to 3.9)	5 (–107 to 117)
2009	–0.1 (–5.6 to 5.3)	–4 (–166 to 159)
50–64		
2003	3.0 (–1.9 to 7.8)	33 (–21 to 87)
2004	9.8 (6.2 to 13.3)	111 (71 to 151)
2005	–1.8 (–5.6 to 2.0)	–21 (–64 to 23)
2006	–4.7 (–8.6 to –0.9)	–56 (–102 to –10)
2007	14.5 (10.9 to 18.2)	176 (132 to 221)
2008	–2.1 (–5.9 to 1.7)	–26 (–73 to 21)
	0.5 (–5.0 to 5.9)	6 (–63 to 75)
65–79		
2003	23.5 (18.7 to 28.4)	154 (122 to 186)
2004	15.6 (12.1 to 19.2)	103 (80 to 126)
2005	–22.1 (–25.9 to –18.4)	–147 (–172 to –122)
2006	–29.3 (–33.2 to –25.5)	–196 (–222 to –171)
2007	25.3 (21.7 to 28.9)	172 (147 to 197)
2008	16.5 (12.8 to 20.3)	114 (88 to 140)
2009	–7.4 (–12.9 to –2.0)	–53 (–91 to –14)
>80		
2003	186.2 (181.4 to 191.1)	425 (414 to 436)
2004	141.3 (137.7 to 144.8)	333 (325 to 342)
2005	–104.7 (–108.5 to –100.9)	–257 (–266 to –247)
2006	–32.6 (–36.4 to –28.7)	–82 (–92 to –73)
2007	53.1 (49.4 to 56.7)	140 (130 to 149)
2008	180.1 (176.4 to 183.9)	492 (482 to 502)
2009	–131.6 (–137.1 to –126.2)	–371 (–387 to –356)

*Rate per 100,000 persons. CI, confidence interval.

**Table 3:** All-age differences between observed and baseline all-cause death rates* and counts, and predominant influenza virus strains, by year, New South Wales, Australia, January 2003–September 2009

Year	Crude rate (95% CI)	Standardized† rate (95% CI)	No. (95% CI)	Predominant strain(s)
2003	10.0 (7.3 to 12.6)	11.1 (6.3 to 16.0)	666 (488 to 843)	A/Fujian/411/2002 (H3N2)-like	
2004	7.2 (5.3 to 9.1)	8.0 (4.5 to 11.6)	482 (352 to 612)	A/Fujian/411/2002 (H3N2)-like	
2005	–6.4 (–8.4 to –4.3)	–6.9 (–10.6 to –3.1)	–432 (–571 to –294)	A/California/7/2004 (H3N2)-like	
2006	–4.3 (–6.4 to –2.2)	–4.5 (–8.3 to –0.6)	–291 (–433 to –149)	B/Malaysia/2506/2004-like (Victoria lineage)	
2007	7.3 (5.3 to 9.2)	7.5 (3.8 to 11.1)	502 (366 to 638)	A/Brisbane/10/2007 (H3N2)-like, A/Solomon Islands/3/2006 (H1N1)-like	
2008	8.6 (6.5 to 10.6)	8.8 (5.0 to 12.5)	600 (457 to 742)	B/Florida/4/2006-like (Yamagata lineage)	
2009	–6.0 (–8.9 to –3.1)	–6.0 (–11.5 to –0.6)	–423 (–630 to –217)	A/California/7/2009 (H3N2)-like, A/Brisbane/10/2007 (H3N2)-like	

*Rates are per 100,000 population. CI, confidence interval. †Age standardized by using the 2009 mid-year age-specific population estimates as the standard population.

In years with all-age increases above baseline, the greatest increases occurred in persons >80 years of age, from 53.1 (95% CI 49.4–56.7) per 100,000 in 2007 to 186.2 (95% CI 181.4–191.1) per 100,000 in 2003. Death rates in persons 65–79 years of age also significantly increased during the same years, from 15.6 (95% CI 12.1–19.2) per 100,000 in 2004 to 25.3 (95% CI 21.7–28.9) per 100,000 in 2007. Death rates were significantly increased in persons 50–64 years of age only in 2004 and 2007. Death rates for all other age groups did not differ significantly from baseline (Table 2), but CIs were wide.

In years with significantly increased all-age death rates, estimated excess deaths ranged from 482 (95% CI 352–612) in 2004 to 666 (95% CI 488–843) in 2003. Except during 2007 in which the count difference was greater both for persons 50–64 and 65–79 years of age, most deaths above baseline were in persons >80 years of age (Table 3).

During May–September 2009, when pandemic (H1N1) 2009 virus first circulated in New South Wales, death rates for persons <65 years of age did not differ significantly from baseline, although the CIs included wide ranges of possible counts. For persons >65 years of age, estimates were significantly lower than expected, particularly in persons >80 years of age, in which the estimated count difference was 371 (95% CI 356–387) below baseline (Table 2). Only in 2005 were the overall estimates further below baseline, but the difference between the 2 years was not significant (Table 3).

Repeating the modeling separately for each age group led to a median difference of 0.5 deaths/100,000 population between the age-specific rate differences using this method and our original method. The mean difference was 3.9/100,000 (range 0.0–29.4/100,000); larger differences occurred in the older age groups where estimates were larger.

We also repeated the original analysis after removing observations from the May–September period in each year and using ordinary linear regression rather than robust regression. Annual all-age estimates of the difference between observed and baseline all-cause death rates were an average 19.8/100,000 greater than those estimated by using robust regression. The year 2003 showed the highest increase above the baseline of 2,096 deaths (31.4/100,000). The year 2009 showed the smallest difference with 594 additional deaths (8.4/100,000) above baseline.

## Discussion

In New South Wales, during May–September 2009, the epidemic of pandemic (H1N1) 2009 during the first Southern Hemisphere winter after its emergence was associated with a decline in rates of all-cause deaths relative to seasonal expectation. The all-age reduction was similar to that of 2005. Despite a small apparent peak in weekly death rates for persons 50–64 and possibly 0–19 years of age that coincided with the peak in influenza activity in 2009, overall May–September differences in 2009 did not differ significantly from zero in persons <65 years of age and were significantly lower than expected in persons >65 years of age. This is consistent with the epidemiologic evidence from reports from Australia (*12**,**19**,**20*) and internationally (*21*) that suggest that persons >60 years of age were relatively protected from infection. Reduced susceptibility in older age may be due to past exposure either through natural infection or vaccination to a similar H1 strain or a strain that provided cross-protective immunity (*22*). Previous pandemics also have spared the older population (*23*). The differences in 2009 for younger persons that were close to zero reflect the limited statistical precision of the indirect method we used rather than reflecting an actual death rate from influenza close to zero. This results from the combination of our relatively small population size and low overall death rates for younger persons.

The apparently low influenza-related mortality in 2009 contrasts with the substantially higher-than-usual demand for emergency department, general inpatient, and intensive care services, particularly among persons <60 years of age (*12*). This could reflect high attack rates for younger persons but fatal outcomes in a small proportion because of greater resilience in younger persons or intensive treatment in younger persons who have serious complications (*24*). The small peak in death rates for persons 50–64 years of age in 2009 (Figure A1) is consistent with the increased relative risk for admission to intensive care units and death in that age group among persons with confirmed pandemic strain infection (*12*). This may reflect increasing prevalence with age of risk factors for death tempered in persons <65 years of age by reduced susceptibility to infection.

In New South Wales, during May–September 2009, with intensive case ascertainment, 51 deaths with confirmed pandemic (H1N1) 2009 virus infection were reported. Most occurred in persons <60 years of age, and one fourth of infected persons were >70 years of age (*25*). Even though these figures cannot be directly compared with our indirect estimates, the count for younger persons would be within the upper range of CIs we estimated in younger age groups. For older age groups, we estimated fewer deaths than baseline, and CIs did not include positive counts. This finding suggests that background noninfluenza death rates may have been lower than usual or strain replacement by the pandemic virus led to lower death rates from nonpandemic influenza later in the season. The Figure A1 does show unusually low death rates for elderly persons late in the 2009 season.

In Australia, seasonal influenza vaccination is offered free to all persons >65 years of age and to younger persons in high-risk groups. In persons >65 years of age, vaccination levels in our state were >70% during 2003–2007 (*26*). We therefore would expect influenza-related mortality to reflect the innate virulence of the circulating virus strains, preexisting population immunity from past exposure to related influenza strains, and the degree of mismatch between the circulating strain and the available influenza vaccine.

The emergence of the Fujian strain in the Northern Hemisphere winter of 2002–03 led to widespread outbreaks. These outbreaks would explain the greatest relative increase in mortality that we observed in 2003. In that year, antigenic match to the available vaccine was poor, and a matching strain was unable to be included in the Southern Hemisphere vaccine until 2004 (*27**–**29*). In 2004, further antigenic drift occurred away from this vaccine strain (*30*) which may explain the continuing increased mortality in 2004, despite the apparently low level of influenza circulation generally.

In 2005, a variant of the Fujian strain, the California 2004 H3N2 strain, predominated in New South Wales. Mortality did not increase, possibly because of cross-protection from the A/Wellington/1/2004 (H3N2)–like strain included in the 2005 Southern Hemisphere vaccine (*31*). In 2006, mortality also did not increase. This was a relatively mild epidemic year in Australia, with the B Malaysia strain, which was included in the local 2006 vaccine (*32*), predominating.

The 2007 epidemic in Australia, in which we next observed substantially increased mortality, including a distinct peak in persons 50–64 years of age, and the 2007–08 epidemic in the United States were relatively severe. These epidemics caused unusually high illness and death in young children (*33**,**34*), consistent with the antigenic drift away from both the 2007 Southern Hemisphere subtype H3N2 and H1N1 vaccine strains (*33*).

In 2008, we observed the second highest estimate of increased mortality of the years we studied. This finding is somewhat surprising because, compared with influenza A (H3N2), influenza B is uncommonly associated with increased mortality (*35*). Yet, the double peak in influenza isolates also was evident in the all-cause and P&I mortality curves for persons >80 years of age in 2008 (Figure A1). Morbidity appeared relatively low in 2008 in our state, but influenza B strains dominated locally and nationally (*19**,**36*). The New South Wales strain data received from the national influenza collaborating center indicated that most influenza B strains of both Yamagata and Victoria lineages from New South Wales in 2008 showed low reactivity to reference strains, suggesting antigenic drift. This could have led to reduced effectiveness of the 2008 and 2007 vaccines, which alternately included a B strain from each lineage.

The alternative analyses we conducted to check the plausibility of our results did not alter our conclusions. Fitting the model separately to each age group made little difference. However, our modeling approach with age group included in a single model ensured consistency of the age-based estimates with all-age estimates. Using a more traditional Serfling approach with exclusion of cooler month data did not change our original conclusion that, in the 2009 pandemic year, influenza-related mortality was relatively low compared with recent influenza seasons. However, this approach led to possibly excessive estimates of influenza-associated mortality. This more conventional method may be overly sensitive to choices made on which periods to exclude. On the other hand, the method we used may have been overly conservative. Nevertheless, the age-specific rate differences we observed using the robust regression method in years with excess influenza activity were broadly similar to those in recent studies in Norway (*37*), the Netherlands (*38*), Italy (*8*), and Canada (*39*) that used various methods.

A limitation of this study was the relatively short time series used. Nevertheless, the number of years included was sufficient to provide a reasonable comparison with the year of primary interest, 2009. The modeling approach does not account for other time-varying factors that might influence death rates during the year, such as other circulating pathogens and meteorologic factors. An implicit assumption in the Serfling method is that the magnitude and timing of the background seasonal mortality pattern is rigidly consistent from year to year, but in reality noninfluenza factors vary from year to year and within years, and the rigidity of the Serfling model would therefore vary in its successful distinction between influenza and noninfluenza mortality. On the other hand, using more flexible models has the risk of following rather than excluding changing mortality associated with influenza activity.

The arrival of pandemic (H1N1) 2009 virus in New South Wales during winter 2009 was associated with a decline in all-cause mortality compared with the usual seasonal pattern. This lower mortality may reflect the relatively low virulence of the virus in most persons infected; the reduced susceptibility of older age groups, who usually are most at risk for complications and death from influenza infection; the success of public health measures, including intensive deployment of the antiviral medication stockpile, the high quality of healthcare available in Australia, or a combination of these factors.

## Supplementary Material

Technical Appendix Robust Regression Model, Confidence Intervals for the All-Age Predicted Baseline Rates, and Sensitivity Analyses
