# Supplementary material for: All-Cause Mortality during First Wave of Pandemic (H1N1) 2009, New South Wales, Australia, 2009
Source: Emerg Infect Dis. 2010 Sep;16(9):1396–402. doi: 10.3201/eid1609.091723 (PMC3294965; doi:10.3201/eid1609.091723)
Supplement: Technical Appendix — Robust Regression Model, Confidence Intervals for the All-Age Predicted Baseline Rates, and Sensitivity Analyses [file 09-1723-Techapp_2p.pdf]

Article DOI: 10.3201/eid1609.091723

All-Cause Mortality during First Wave of Pandemic (H1N1) 2009, New South Wales, Australia, 2009

Technical Appendix

Robust Regression Model

The model was

$$
\begin{aligned}
&Expected(death\ rate) \\
&\quad = \beta_1 + \beta_2 t + \beta_3 t^2 + \beta_4 \sin\left(\frac{2\pi t}{52.167}\right) + \beta_5 \cos\left(\frac{2\pi t}{52.167}\right) \\
&\quad + \beta_6(age) + \beta_7(age \times t) + \beta_8(age \times t^2) \\
&\quad + \beta_9\left(age \times \sin\left(\frac{2\pi t}{52.167}\right)\right) + \beta_{10}(age \times \cos(\frac{2\pi t}{52.167}))
\end{aligned}
$$

where *t* is the week number and *age* is categorical age (0–19, 20–49, 50–64, 65–79, $\geq$80 y). The sine and cosine functions of time are the harmonic terms, which define the phase and amplitude of the annual seasonal oscillations in death rates. The β parameters are estimated by the model fitting and reflect the relative contribution of each factor to the expected death rate. These models are also called trigonometric models (*1*).

PROC ROBUSTREG in SAS version 9.1, using the default M-estimation method, was used to fit the model (*2*). M-estimation is useful when distorted (or contaminated values) are in the response direction only (*3*). In our case, the response is the death rate, which is contaminated by influenza epidemics.

Confidence Intervals for the All-Age Predicted Baseline Rates

For each week of the study, to obtain confidence intervals of the total of the age-specific predicted baseline rates, we first had to calculate the standard error of the sum of the age-specific

baseline rates. This was performed by using the standard formula for estimating the standard error of the sum of several independent estimates, which involved taking the square root of the sum of the squared standard errors of the age-specific estimates. Multiplying the result by 1.96 and adding it to the total weekly predicted baseline rate provided the upper 95% confidence limit. Subtracting provided the lower limit. The standard errors of the age-specific weekly baseline estimates were obtained from the model output file. The standard error of the mean predicted value option of the OUTPUT statement in PROC ROBUSTREG was used to have these included.

Sensitivity Analyses

For separate analysis of individual age groups, PROC ROBUSTREG in SAS version 9.1 was used to implement the following model within each age group:

$$Expected(death\ rate) = \beta_1 + \beta_2 t + \beta_3 t^2 + \beta_4 \sin(\frac{2\pi t}{52.167}) + \beta_5 \cos\left(\frac{2\pi t}{52.167}\right)$$

For analysis using a more traditional Serfling approach, PROC GENMOD in SAS version 9.1 was used with DIST=NORMAL in the MODEL statement for an assumption of normally distributed residuals. The first model with age and age interactions above was applied after setting the all-cause death rates for weeks ending during May–September to missing values.

References

1. Graybill FA. Theory and application of the linear model. Belmont (CA): Wadsworth; 1976

2. Huber PJ. Robust estimation or a location parameter. Ann Math Stat. 1964;35:73–101.

3. SAS Institute. SAS/STAT(R) 9.2 user’s guide. 2nd ed. Cary (NC): SAS Institute Inc.; 2009
